# Uterine Arteriovenous Fistula with Concomitant Pelvic Varicocele: Endovascular Embolization with Onyx-18®

**DOI:** 10.1155/2017/3548271

**Published:** 2017-11-22

**Authors:** Francesco Giurazza, Fabio Corvino, Andrea Paladini, Antonio Borzelli, Domenico Scognamiglio, Giulia Frauenfelder, Giuseppe Albano, Fabio Sirimarco, Raffaella Niola

**Affiliations:** ^1^Interventional Radiology Department, Antonio Cardarelli Hospital, Via A. Cardarelli 9, 80100 Naples, Italy; ^2^Maggiore Hospital of Lodi, Viale Savoia 1, 26900 Lodi, Italy; ^3^Radiology Department, Campus Bio-Medico University, Via Alvaro Del Portillo 200, 00100 Rome, Italy; ^4^Gynaecology and Obstetrics Department, Antonio Cardarelli Hospital, Via A. Cardarelli 9, 80100 Naples, Italy

## Abstract

Uterine arteriovenous fistulas are rare and acquired causes of life-threatening vaginal bleeding. They usually present with intermittent menometrorrhagia in young patients in childbearing age with history of gynecological procedures on uterus. Traditional management is hysterectomy; endovascular embolization represents nowadays an alternative strategy for patients wishing to preserve fertility. Here, the endovascular approach to a 29-year-old woman affected by severe menometrorrhagia caused by a uterine arteriovenous fistula with a concomitant pelvic varicocele is reported; a bilateral uterine arteries embolization with Onyx-18 (ev3, Irvine, CA, USA) has successfully resolved the fistula with clinical success.

## 1. Introduction

A uterine arteriovenous fistula (AVF) is a rare acquired cause of life-threatening vaginal bleeding; it may be the result of trauma or surgical intervention or may be acquired in the setting of a preexisting pathologic uterine process [1].

Here, the endovascular approach to a 29-year-old woman affected by severe menometrorrhagia caused by an AVF with a concomitant pelvic varicocele is reported; the lesion has been resolved with a bilateral uterine arteries embolization (UAE) by Onyx-18 (ev3, Irvine, CA, USA) injection.

## 2. Case Report

A 29-year-old woman presented with severe vaginal bleeding in emergency room with a value of 7 g/dl of hemoglobin.

She reported episodes of severe menometrorrhagia during the previous 4 months.

Her clinical history presented multiple interventions on the uterus: a hysteroscopic ablation 5 years previously to remove a pedunculated lesion (histological exam: pedunculated leiomyoma), a successful pregnancy with cesarean Section 3 years previously, and a uterine curettage to remove an ectopic cervical pregnancy 4 months before hospital recovery and just before clinical presentation.

**Figure 1:**
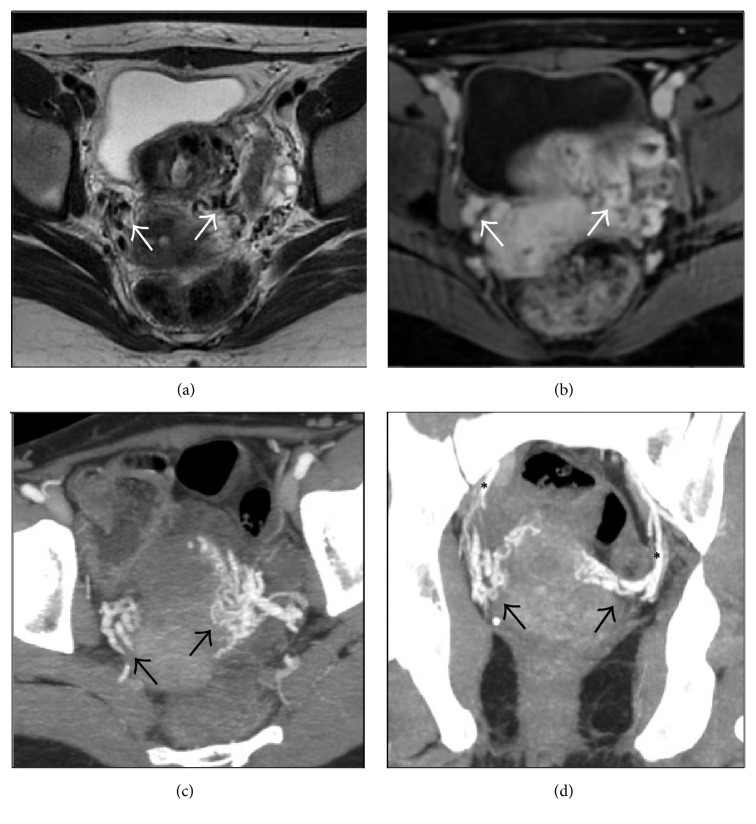
MR ((a) and (b)) and CT ((c) and (d)) before the embolization. (a) T2-weighted MR in axial plane; (b) contrast-enhanced T1-weighted MR in axial plane; arterious contrast-enhanced CT with maximum intensity projection in axial (c) and coronal planes (d). Multiple ectatic and serpiginous anomalous vessels adjacent to the uterine wall bilaterally are appreciable at MR and CT imaging; these are indicated by white arrows in (a) and (b) and black arrows in (c) and (d). In (d), uterine arteries, indicated by black asterisks, are in direct connection with the anomalous vessels of the uterine wall.

During the hospital stay, she underwent a suprapubic ultrasound (US) examination revealing a high flow vascular lesion involving the uterine wall; a contrast-enhanced Magnetic Resonance (MR) (Figures 1(a) and 1(b)) showed multiple ectatic and serpiginous anomalous vessels adjacent to the uterine wall bilaterally. Vessels enhancement is appreciable after contrast injection in Figure 1(b); a contrast-enhanced Computed Tomography (CT) (Figures 1(c) and 1(d)) showed an abnormal arterious vascular network involving the uterus with concomitant pelvic varicocele; both uterine arteries supplied the lesion with aberrant venous outflow.

The beta human Chorionic Gonadotropin (hCG) test was negative.

According to laboratory and radiological data, considering even the previous multiple uterine interventions, the diagnosis of uterine AVF was proposed.

Because of the young age of the patient and the will of future pregnancies, conventional surgery consisting of hysterectomy was avoided and a conservative endovascular approach was considered as first option.

**Figure 2:**
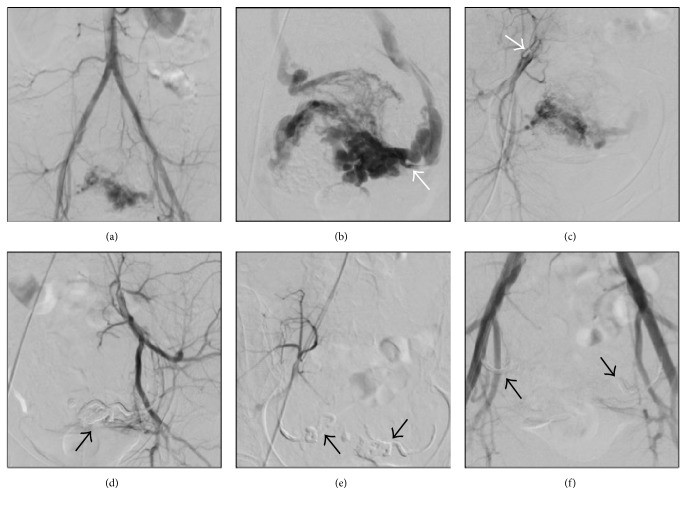
Procedural imaging and digital subtraction angiography. (a) Pelvic angiogram after 5Fr pigtail catheter positioning in abdominal aorta showing the vascular lesion in arterious phase injection. (b) Selective left uterine arteriography by 5Fr Cobra catheterization positioned crossing the aortic carrefour (white arrow): ectatic and aberrant arterious vessels with concomitant ectatic and serpiginous direct venous outflow, confirming the AVF diagnosis. (c) Selective right uterine arteriography by 5Fr Robert Uterine Curve catheterization (white arrow): involvement of the right uterine artery in terms of inflow to the AVF. ((d) and (e)) Left and right postembolization hypogastric artery angiography, respectively: complete occlusion of the uterine arteries after Onyx-18 injection and preservation of the other hypogastric branches; the Onyx-18 cast is indicated by the black arrows. (f) Final pelvic angiogram: the AVF is no more appreciable after embolization with Onyx-18 (black arrows indicate the casts).

The patient underwent a bilateral superselective UAE (Figure 2); the procedure was performed with a monolateral access by positioning a 5Fr sheath into the right common femoral artery. First, an aortogram and a pelvic angiogram using a 5Fr pigtail catheter were acquired and confirmed the high-flow AVF, excluding the involvement of arteries originating from body districts other than the hypogastric trunk (Figure 2(a)). A selective hypogastric arteriogram was obtained bilaterally, using a 5Fr Cobra curve for the (left) contralateral artery (Figure 2(b)) and a 5Fr Robert Uterine Catheter Curve for the (right) homolateral artery (Figure 2(c)): these demonstrated that both uterine arteries refurnished the fistula, with a major component from the left side; no other vessels were involved, confirming the findings of CT and MR.

Based on the diagnostic angiograms, the embolization of both uterine arteries was planned during the same session.

The most distal portion of the uterine arteries was superselectively reached with a microcatheter using a coaxial technique; the 5Fr catheter was positioned at the origin of the uterine artery and the microcatheter was advanced distally over the horizontal segment of the uterine artery; the diagnostic angiogram showed the venous outflow directly communicating with arterious inflow, confirming the fistula diagnosis.

The embolization was performed using a definitive liquid agent (Onyx-18) (Figures 2(d) and 2(e)); first, an Onyx cast was injected around the microcatheter tip to avoid nontarget reflux and then the embolizing Ethylene Vinyl Alcohol copolymer was slowly injected and pushed forward by Dimethyl Sulfoxide.

The final angiographic control (Figure 2(f)) showed completed embolization of both uterine arteries up to the FAV nidus with consequent disappearance of the venous outflow.

The patient was monitored for 5 days with nonsteroid anti-inflammatory medication because of a mild postembolization syndrome and then was dismissed.

**Figure 3:**
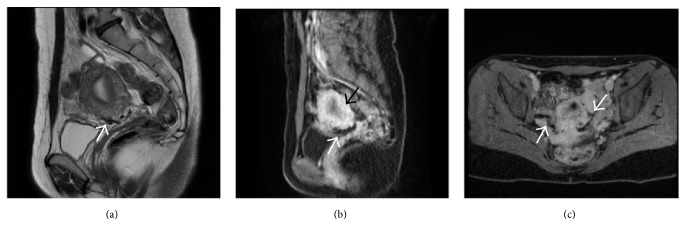
Postoperative MR 30 days after the embolization. (a) T2-weighted MR in sagittal plane: uterine wall preservation with serpiginous Onyx-18 cast (white arrow) into the left uterine artery. ((b) and (c)) Contrast-enhanced T1-weighted MR, sagittal and axial planes, respectively: preserved uterine wall perfusion (black arrow in (b)) without enhancement of the uterine arteries because of the Onyx-18 embolization (white arrows in (b) and (c)).

The MR follow-up after 30 days showed resolution of the fistula with consequent disappearance of the varicocele; uterine vascularization was preserved without signs of necrosis (Figure 3).

No other episodes of menometrorrhagia occurred during the 3-month clinical follow-up and the period returned to normal 1 month after embolization.

## 3. Discussion

Uterine arteriovenous malformations can be congenital or acquired; the latter are named AVFs and are more common than congenital lesions that on the other hand should be properly named as arteriovenous malformations (AVM) [1].

The correct diagnosis of uterine AVF is based on negative hCG test and imaging of abnormal uterine vascular network with tortuous and serpiginous vessels [2]. Clinically, these are symptomatic patients for menometrorrhagia: bleeding is intermittent and torrential due to the differential pressure gradient across arterial and venous systems [2]. Women are typically in childbearing age with a history of uterine trauma secondary to gynecological procedures [3].

The traditional treatment is hysterectomy [4]; however, endovascular embolization represents nowadays an alternative strategy for patients wishing to preserve fertility.

The first conservative treatment for uterine AVF has been reported in 1982 [5]; since then few cases of embolization [1–10] have been described.

As for AVF or AVM from other body districts, it is crucial to recognize and embolize all the arterious feeders; typically uterine AVFs do not present with extrauterine involvement and are fed only by uterine arteries [1].

The technical success should always be related to the clinical success because AVF frequently relapses [7]; therefore, long-term clinical and radiological postprocedural follow-up is recommended, almost up to three months.

The risk of uterine necrosis should be considered after definitive UAE; however, the vascularization is usually supplied by the wide uterine collaterals network, as ovarian, spinal, epigastric, and hypogastric arterial branches [11].

Different embolizing agents, as coils, Spongel, particles, and glue alone or in combination, have been proposed [1–10]; overall embolization presents a primary success rate of 61% and secondary success rate of 91% after repeated embolization [1].

Only one paper [2] recently reported Onyx as single agent for the treatment of uterine AVF; in the presented case, embolization with Onyx-18 resolved both the FAV and the concomitant pelvic varicocele after a single procedural session.

There is no consensus about the best embolizing agent and there are also no technical guidelines because uterine AVF is a rare entity; however, endovascular conservative management should be considered as an alternative to hysterectomy in order to preserve fertility in young women wishing for future pregnancies.
